# Bed net care practices and associated factors in western Kenya

**DOI:** 10.1186/s12936-019-2908-6

**Published:** 2019-08-14

**Authors:** Ellen M. Santos, Jenna E. Coalson, Elizabeth T. Jacobs, Yann C. Klimentidis, Stephen Munga, Maurice Agawo, Elizabeth Anderson, Nancy Stroupe, Kacey C. Ernst

**Affiliations:** 1 Mel and Enid Zuckerman College of Public Health, University of Arizona, 1295N Martin Ave, Tucson, AZ 85724 USA; 2 Centre for Global Health Research, Kenya Medical Research Institute, PO Box 1578, Kisumu, Nyanza 40100 Kenya

**Keywords:** Malaria, Kenya, LLIN, ITN, Bed nets, Care and repair

## Abstract

**Background:**

Insecticide-treated nets (ITNs) and long-lasting insecticidal nets (LLINs) are effective for malaria prevention and are designed to provide nearly 5 years of mosquito protection. However, many ITNs and LLINs become damaged and ineffective for mosquito bite prevention within 1 to 2 years in field conditions. Non-adherence to recommended bed net care and repair practices may partially explain this shortened net longevity.

**Methods:**

Using data from a cross-sectional study, a net care adherence score was developed and adherence to net care practices described from two regions of western Kenya. Relationships between attitudes and environmental factors that influence net longevity were measured with adherence to bed net care practices.

**Results:**

While overall care practices are highly adherent particularly in the highlands, practices related to daily storage, washing frequency, and drying location need improvement in the lowlands. Seventy-seven percent of nets in the lowlands were washed < 3 months prior to the survey compared to 23% of nets in the highlands. More nets were dried in the sun in the lowlands (32% of nets) compared to the highlands (4% of nets). Different elements of care are influenced by various malaria attitudes and environmental factors, highlighting the complexity of factors associated with net care. For example, households that learned about net care from community events, that share a sleeping structure with animals, and that have nets used by adult males tend to adhere to washing frequency recommendations.

**Conclusions:**

In western Kenya, many nets are cared for in accordance to recommended practices, particularly in the highlands sites. In the lowlands, demonstrating methods at community events to tie nets up during the day coupled with messaging to emphasize infrequent washing and drying nets in the shade may be an appropriate intervention. As illustrated by differences between the highlands and lowlands sites in the present study, should interventions to improve adherence to bed net care practices be necessary, they should be context-specific.

**Electronic supplementary material:**

The online version of this article (10.1186/s12936-019-2908-6) contains supplementary material, which is available to authorized users.

## Background

Insecticide-treated nets (ITNs) and long-lasting insecticidal nets (LLINs) are effective malaria prevention tools. Notably, ITNs have reduced malaria incidence by half in children under 5 in areas of stable malaria transmission [1]. The primary strategy for malaria prevention in sub-Saharan Africa, according to the World Health Organization (WHO), has become the distribution of LLINs [2]. To achieve their optimal effectiveness, bed nets must be available as a resource (supply), accessible to households (ownership and universal access), used regularly (use) and well maintained to maximize efficacy (care and repair).

While some LLIN distribution programmes have shifted to combinations of continuous and mass distribution models [3], many malaria control programmes still distribute LLIN every 3–5 years, the anticipated optimal lifespan of a LLIN [4]. Initiated in 2005 under President George W. Bush, the President’s Malaria Initiative (PMI) developed a network of monitoring sites to measure realistic LLIN durability and lifespan in home conditions [5, 6]. Studies from these monitoring sites find variable serviceable lifespans generally between 2 and 3 years, though significant damage noticed from as little as 6 months to 4.5 years, with differences by net brand and variations in insecticidal activity and attrition [5, 7–15]. These studies also suggest that inadequate bed net maintenance behaviours contribute to this shortened lifespan [8].

In an effort to standardize terminology for research into bed net maintenance attitudes and practices, the PMI-funded NetWorks project established the phrase “care and repair”. The project developed a conceptual framework of the knowledge, attitudes, intentions, social norms, and behaviours related to care and repair practices [16]. Proper care and repair of LLINs increases their effective longevity, maintaining higher and more stable coverage of vector control between mass community distributions [5, 8, 9]. Recommended LLIN care practices include hanging nets up while not in use, washing gently and infrequently and with no soap or mild soap, drying indoors or in the shade, and repairing holes [17]. Reported adherence to these practices has been mixed, with high adherence to some practices and low adherence to others. A study in Laos examined maintenance behaviours 2–3 years after LLIN distribution and found that 38.2% of households followed the recommended washing frequency, 83% followed drying recommendations, and 32.7% of households with torn LLINs had repaired them [18]. However, the results showed weak associations between maintenance behaviours and malaria knowledge and prevention attitudes [18], obfuscating how educational interventions may be designed to improve adherence. Other studies qualitatively describe how caring for the family and saving money motivate bed net care and repair, though few LLINs in the study area were actually repaired [19, 20]. Perceptions of social acceptability and colour of the LLIN have also been associated with washing frequency, possibly due to social implications of having a dirty net [21–23].

**Fig. 1:**
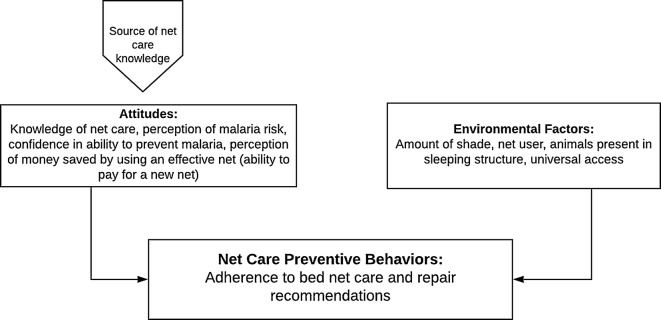
Attitudes and environmental factors related to adherence to net care and repair behaviours (adapted from the NetWorks conceptual framework [16])

These previous studies demonstrate that while social norms and individual perceptions and knowledge influence how LLIN care and repair is conducted, these relationships are complex and vary among communities. Additionally, the NetWorks conceptual framework includes environmental factors such as household attributes that may influence the practicality of adhering to optimal care and repair practices [16]. This study examines associations between care and repair behaviours and attitudes and environmental factors from the NetWorks conceptual framework [16] in western Kenya (Fig. 1). Understanding factors related to appropriate net care and repair behaviours will clarify what messaging or other intervention strategies are needed, and where/how they should be targeted to extend the real-world longevity of LLINs.

## Methods

### Overview

Bed net care practices from a cross-sectional study administered in two regions of western Kenya with differing *Plasmodium* transmission patterns are described. Though the vast majority (95.3%) of nets were LLINs, the term ‘bed net’ or ‘net’ was chosen because the brand or type of all nets could not be verified in the sample. Development of a bed net care adherence score and examined the relationship between overall adherence to care recommendations and net condition. The relationship between adherence and attitudes and environmental elements of the NetWorks conceptual model was examined [16].

### Study design and data collection

Household surveys were administered during a cross-sectional study in western Kenya. Surveys were administered between June and August of 2015 in two sites (highlands and lowlands) of differing *Plasmodium* transmission patterns. Data collected included household, individual, and bed net items encompassing demographic information, malaria history, knowledge and perceptions, and bed net attributes, including ownership, universal access, care, and use. Bed net and household level data were used for the present analyses.

### Study sites

Kapkangani location encompasses the rural highland sublocations of Chepsonoi, Kiborgok, and Tindinyo at an altitude range of 1600–2100 m above sea level and seasonal *Plasmodium* transmission following the rainy season from April to May. In this region, the population is primarily comprised of Kalenjin and Luhya ethnic groups. The primary occupation is rural subsistence agriculture, while some work as casual laborers in the commercial tea industry. Miwani is located on the Kano Plain and includes the rural lowland sublocations of Kabar Central and Kabar West at an altitude of 1200 m above sea level. Transmission is holo-endemic and year-round with seasonal increases. People are primarily of the Luo ethnic group and are subsistence farmers with some employed by area commercial sugarcane and rice growers.

### Household selection

The study site was mapped and enumerated prior to sample selection. Households were randomly selected from an enumerated list. Households were oversampled by 20% and then randomly ordered. Visits were made to each household in subsequent order until the required sample size for each village had been met. Male or female heads of household were approached in person by field team staff for recruitment. Households were included if they had resided in the study area for a period of at least 1 month. Households of all sizes and configurations were included. A household was defined as individuals who regularly eat meals together. Data from 1217 households (n = 640 in Kapkangani and n = 572 in Miwani) were used in the present study.

### Ethical considerations

The current analysis involved secondary analyses of the de-identified data from the primary study. The primary study was approved by the Kenyan Medical Research Institute (KEMRI) (SSC #2810) and the University of Arizona ethical review boards with deferral of primary oversight from the University of Arizona to KEMRI. Informed consent was obtained from all household heads. Parental consent was obtained for children less than 7 years of age, and assent was obtained for those 8 to 17 years of age.

### Bed net care adherence score

Data on each net owned by the household were collected in addition to overall net care practices reported by the household head. A 7-item care scoring system was developed from factors including net storage, washing, and drying. Each net received a score between 0 and 2 for each item and the scores were totaled for an overall net care adherence score, ranging from 0 to 14 in which lower scores indicate better adherence (Table 1). This scoring system was chosen to be more flexible and nuanced than a strict binary system of good-inadequate care. Similar scoring systems are used in cancer prevention literature [24, 25]. The association between net care adherence score with overall net condition was measured. Field staff classified each net as excellent, good, fair, or poor condition in comparison to standard images provided on the survey forms (See Additional file 1).

**Table 1:** Elements of the bed net care adherence scoring system

Score	Storage	Wash freq	Where washed	Soap type	Wash manner	Drying location	Ever retreat*
0 Best practice	Tied up	Never	Washtub or bucket; Never wash	None; Never Wash or Bar soap	Not scrubbed/beaten; Never wash	Indoors; Never wash	Never if LLIN; ever if ITN ≥ 6 months
**1** Moderate practice	Removed	≥ 3 months	Lake	Detergent	–	Outdoors in shade	–
**2** Non-adherent practice	Leave as is	< 3 months	Stream or river	Bleach	Scrubbed or beaten	In the sun	Never if ITN if net age ≥ 6 months, Ever if LLIN

Scores of 0 indicate best practice, scores of 1 indicate moderately adherent practice, and scores of 2 indicate non-adherent practice

*Retreatment is no longer a recommended practice, though households still mentioned the practice, likely from prior experience with ITNs. Retreatment was included to assess knowledge of the practice to determine whether messaging is necessary to discourage retreatment of LLINs

Individual net care elements with ≥ 20% poor adherence (scores of 2) were then selected for further analysis, as these indicate the elements that may need improvement in the study population. Associations of attitudes and environmental factors with adherence to these selected individual net care elements were measured to inform focused interventions.

### Attitudes and environmental factors

*Knowledge of bed net care* is defined as the interviewee’s (household’s) knowledge of particular net care and repair practices taken from an open-ended survey question. Participants were asked what things they should do to take care of their bed net. Whether participants mentioned a particular net care practice indicated knowledge of that practice. Risk perception operates both intra and inter-personally [26].

*Perception of malaria risk* was measured from participant’s perception of the seriousness of malaria for their family (intrapersonal) and for their community (interpersonal). Both of these items are scored on a 5-point likert scale where 1 indicates ‘not at all serious’, and 5 ‘extremely serious’. These items were added together to obtain an overall risk perception score (range 2–10).

*Confidence in the ability to prevent malaria* indicates how well participants felt they are able to protect their family from malaria from a 5-point likert scale where 1 indicates ‘not at all’ and 5 ‘extremely well’.

Household structural factors include both family and physical structures. Family structure includes the *bed net user*, which is a series of binary variables indicating which household members slept under the net. Net user variables measured in years include: child < 5, child 6–18, adult male 19–50, adult female 19–50, adult male 50+, and adult female 50+.

*Source of net care knowledge* includes a series of binary variables indicating where the interviewee learned net care. These variables include: clinic staff, community education session, or during a net distribution.

Self-reported *ability to pay for a bed net* is a binary variable where ‘yes’ indicates the household could afford a bed net if one was not provided for free. The ability to pay for a net may influence how well nets are cared for depending if it is easy or difficult to obtain a new net.

Physical structural factors include *universal access*, defined as households that meet the WHO recommendation of one net for every two household members, *amount of shade in the house compound*, defined as no shade, little, half-shaded, or very shaded, and *animals sleep in structure at night*, a binary variable where ‘yes’ indicates an animal shares the sleeping structure space at night.

### Statistical analyses

Stata 12.1 (College Station, TX) was used for all analyses with α = 0.05 at the bed net level. Due to the clustered, dependent nature of the data (multiple nets per household), Generalized Estimating Equations (GEEs) were used to measure the association between net condition and overall net care adherence score, and to measure the associations between the identified key independent variables and binary bed net care adherence elements. Binary care adherence elements were used to assess associations with non-adherent practices, thus best and moderate practices were grouped together to serve as the reference category. GEEs are flexible and ideal for analyzing dependent data that do not meet the assumptions of classical regression techniques [27, 28].

These models can also be used for ordinal discrete data [29]. The GEE parameters used for adherence score analyses were adjusted for bed net age and included Gaussian family, identity link function, exchangeable correlation structure, and robust standard errors. The parameters used for individual net care element analyses included binomial family, identity link function, exchangeable correlation structure, and robust standard errors. GEE models assessing washing frequency were adjusted for net age because this element was measured as “when the net was last washed”. Because the highlands and lowlands differed on nearly all demographic characteristics, level of malaria endemicity, and history of LLIN distributions and community education programmes, GEEs were run separately for each site.

## Results

Net ownership was greater in the lowlands where 98% of households owned at least one net compared to 68.6% of households in the highlands (Table 2). Most households had one sleeping structure, though households in the highlands tended to have both more individuals in the household as well as more sleeping structures than in the lowlands, where 29.6% vs. 10.2% had more than one structure, respectively. Females are primarily responsible for net care [30], thus it is important to understand attributes of female household heads. In both sites, most female household heads had some primary education or have completed primary education (Table 2).

**Table 2:** Household characteristics of study sample by site

	Highlands (643 households) *N* (%)	Lowlands (574 households) *N* (%)
People in household		
1–3	241 (37.5)	382 (66.6)
4–6	296 (46.0)	169 (29.4)
≥ 7	106 (16.5)	23 (4.0)
Sleeping structures in household		
1	453 (70.5)	516 (89.9)
2	157 (24.4)	50 (8.7)
3	27 (4.2)	7 (1.3)
4	5 (0.8)	1 (0.2)
5	1 (0.2)	0
Nets per household		
0	203 (31.6)	11 (1.9)
1	167 (26.0)	318 (55.4)
2	154 (24.0)	185 (32.2)
3	86 (13.4)	45 (7.8)
4	28 (4.4)	14 (2.4)
≥ 5	5 (0.8)	1 (0.2)
Education of the female household head		
None	55 (8.6)	22 (3.8)
Some primary	261 (40.6)	200 (34.8)
Completed primary	152 (23.6)	190 (33.1)
Some secondary	66 (10.3)	57 (9.9)
Completed secondary	30 (4.7)	39 (6.8)
Any tertiary	20 (3.1)	8 (1.4)
No female household head	42 (6.5)	50 (8.7)

### LLIN care adherence

**Fig. 2:**
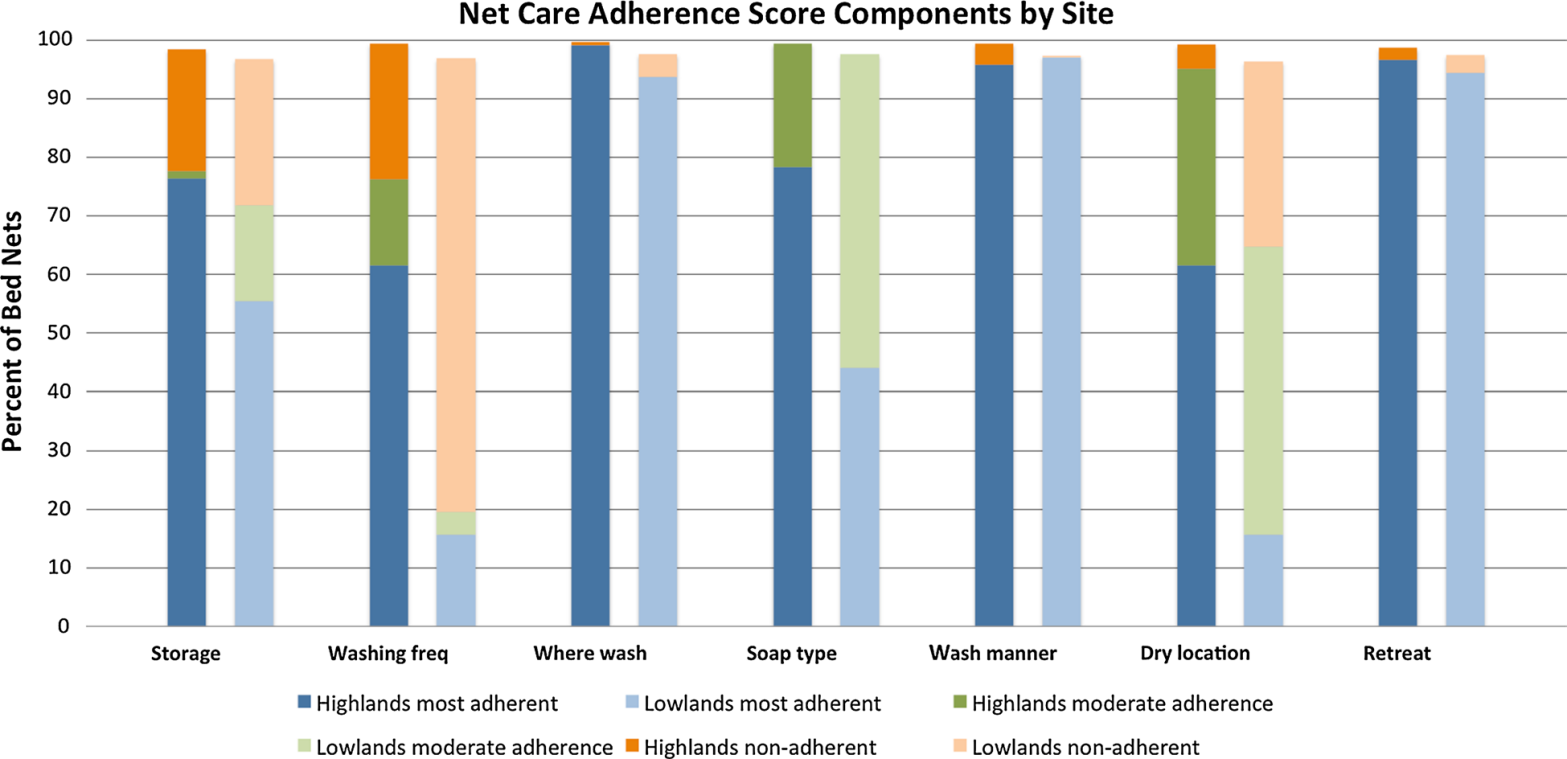
Individual bed net care adherence score components by site. Scores for components in the highlands are the left-sided, bold coloured bars, and the scores for components in the lowlands are the right-sided, pale coloured bars

Bed nets in the highlands had better overall care adherence scores compared to the lowlands. Adherence in the highlands was quite good, where nets had a median adherence score of 2, compared to scores in the lowlands, that while still good, had a lower adherence score of 4 (possible range 0–14) (Table 3). When each care element was measured individually, 2 elements in the highlands and 3 in the lowlands had inadequate adherence (< 80% of nets cared for according to recommendations) (Fig. 2). Adherence to washing location (bucket vs. river or stream), washing manner (gently washed or scrubbed/beaten), and soap type were adequate in both sites and excluded from further analyses. It appears that LLINs were not retreated. However < 80% of nets met adherence recommendations for daily storage, washing frequency, and drying location in at least one of the sites (highlands or lowlands). Frequent net washing is not recommended, and nets in the lowlands were washed more frequently (77% < 3 months) than the highlands (23% < 3 months). In the lowlands, 32% of nets were inappropriately dried in the sun compared to only 4% in the highlands (Fig. 2).

**Table 3:** Distributions of net care adherence and attitudes and environmental factors

	Highlands^CDE^	Lowlands^CDE^	p-value^A^
	Median (range) or # (%)	Median (range) or # (%)	
Overall net care adherence score^F^	2 (0–8)	4 (0–9)	< 0.001*
Overall net condition			< 0.001*^G^
Excellent	522 (59.9)	594 (67.2)	
Good	227 (26.1)	162 (18.3)	
Fair	98 (11.3)	85 (9.6)	
Poor	18 (2.1)	23 (2.6)	
Knowledge and attitudes			
What kinds of things should you do to take care of your bed net?^C^			
Tie up in the morning	177 (27.6)	94 (16.4)	< 0.001*
Don’t wash or wash rarely	25 (3.9)	44 (7.7)	0.005*
Don’t use detergent	135 (21.1)	37 (6.5)	< 0.001*
Don’t beat on the rocks	149 (23.2)	48 (8.4)	< 0.001*
Don’t hang in the sun	382 (59.6)	281 (49.0)	< 0.001*
Re-treat with insecticide	213 (33.2)	271 (47.2)	< 0.001*
Tie up all holes	166 (25.9)	418 (72.8)	< 0.001*
Perception of malaria risk^C^ (2 = low, 10 = high)	6 (2, 10)	7.5 (2, 10)	< 0.001*^B^
Confidence in ability to prevent malaria^C^ (likert scale)	2 (1, 5)	3 (1, 5)	<0.001*
Environmental factors			
Household family structure: net user (years)^D^			
Child < 5	219 (25.0)	232 (26.2)	0.386
Child 6–18	332 (38.0)	312 (35.3)	0.387
Adult male 19–50	169 (19.3)	218 (24.7)	0.003*
Adult female 19–50	332 (38.0)	337 (38.1)	0.386
Adult male > 50	61 (7.0)	58 (6.6)	0.811
Adult female > 50	113 (12.9)	101 (11.4)	0.416
Ability to pay for a net^C^	441 (68.8)	494 (87.1)	< 0.001*
Source of net care knowledge^C^			
Clinic	328 (37.5)	372 (42.1)	0.127
Community event	176 (20.1)	340 (38.5)	< 0.001*
Net distribution	179 (20.5)	3 (0.34)	< 0.001*
Universal Access (1 net:2 members)	239 (37.2)	414 (72.1)	< 0.001*
Amount of shade in house compound^C,G^			< 0.001*
No shade	60 (9.3)	106 (18.5)	
Little shade	321 (49.9)	255 (44.4)	
Half shaded	229 (35.6)	159 (27.7)	
Very shaded	27 (4.2)	38 (6.6)	
Sleeping structure has Earth floor^E^			< 0.001*
Earth floors	916 (73.5)	893 (87.0)	
No earth floors	331 (26.5)	134 (13.0)	
Animals present in sleeping structure at night^E^			0.004*
Animals present	164 (13.2)	179 (17.4)	
No animals present	1083 (86.8)	848 (82.6)	

^A^Two sample test of proportions

^B^Nonparametric equality of medians test

^C^Household level data (highlands *n *= 641, lowlands *n *= 574)

^D^Bed net level data (highlands n = 874, lowlands n = 884)

^E^Sleeping structure level data (highlands n = 1247, lowlands n = 1027), *statistically significant at alpha 0.05

^F^Lower scores indicate better net care and repair adherence practices

^G^Pearson Chi square test

### Attitudes and environmental factors

Households in both sites were most knowledgeable about proper drying practices (drying nets out of the sun). In the highlands, 59.6% of households specifically mentioned nets should not be dried in the sun compared to 49.0% of households in the lowlands (Table 3). This knowledge translated to practice in the highlands (4% of nets dried in the sun), but less so in the lowlands (32% of nets dried in the sun) (Fig. 2). Notably in both sites only 25 (3.9%) of households in the highlands and 44 (7.7%) in the lowlands mentioned they should not wash their nets or wash them infrequently, suggesting that messaging regarding proper washing frequency has not been well communicated in these sites (Table 3). Perception of malaria risk and reported confidence in ability to prevent malaria were both greater in the lowlands compared to the highlands (Table 3).

Nearly a quarter of nets were used by at least one child < 5 years in both sites. The distribution of net users is similar in both sites, though more nets were used by males 19–50 years in the lowlands compared to the highlands. Significantly more households in the lowlands (87.1%) reported the ability to afford a bed net compared to households in the highlands (68.8%) (Table 3).

Most households learned about net care and repair practices from clinics in both sites. Approximately 20% of households learned from community events and nearly 20% from net distributions in the highlands, in contrast with the lowlands where 38.8% learned from community events and only 0.34% learned from a net distribution.

### Factors associated with net care adherence

There was no association between net condition and overall net care adherence score in the highlands (Table 4). However in the lowlands, poor net condition was associated with worse overall net care adherence score after adjustment for net age (Table 4).

**Table 4:** GEE results of association between net condition and overall net care adherence score, adjusted for net age

	Highlands		Lowlands	
	Coefficient	95% CI	Coefficient	95% CI
Overall net condition				
Excellent	Ref		Ref	
Good	0.48	− 0.34, 1.29	0.46	− 0.45, 1.36
Fair	0.61	− 0.16, 1.38	0.64	0.42, 0.86
Poor	− 0.27	− 1.75, 1.22	0.96	0.50, 1.41

Associations measured using GEE models with net care adherence as an ordinal variable

Different attitudes and environmental factors were associated with elements of net care in different ways (Table 5). Knowledge of net care, and nets used by children < 5 years, children 6–18 years, adult males 19–50 years, and adult males > 50 years were associated with better adherence (lower scores). Nets used by adult females > 50 years were associated with worse adherence (higher scores). Many independent variables had positive associations with some elements of the net care adherence score, and negative associations with others, illustrating the complexity of the factors that influence net care behaviours.

**Table 5:** Univariate GEE models measuring associations between attitudes and environmental factors with binary net care practices

Attitudes and environmental factors	Highlands		Lowlands		
	Storage	Wash Freq^A^	Storage	Wash Freq^A^	Dry location
Knowledge of appropriate care			*0.86 (0.80, 0.92)*		*0.86 (0.80, 0.92)*
Perception of malaria risk			1.03 (1.02, 1.05)		
Confidence to prevent malaria	1.06 (1.02, 1.11)		1.08 (1.04, 1.13)		
Net user					
Child < 5	*0.92 (0.86, 0.99)*		*0.93 (0.87, 0.98)*		
Child 6–18			*0.91 (0.85, 0.97)*		
Adult male 19–50					
Adult female 19–50					
Adult male > 50		*0.93 (0.91, 0.96)*			
Adult female > 50	1.27 (1.14, 1.40)				
Ability to buy net			1.10 (1.01, 1.20)	1.15 (1.04, 1.29)	
Source of net care knowledge					
Clinic			*0.87 (0.82, 0.93)*		1.10 (1.02, 1.19)
Community event	*0.87 (0.81, 0.93)*	*0.93 (0.90, 0.96)*	1.24 (1.17, 1.33)	1.07 (1.01, 1.13)	*0.87 (0.81, 0.94)*
Net distribution	1.20 (1.09, 1.33)				
Universal Access (1 net: 2 people)			1.11 (1.03, 1.19)		
Number of trees around compound	N/A	N/A	N/A	N/A	
Sleeping structure attributes					
Earth floors	1.07 (1.01, 1.14)				1.14 (1.04, 1.26)
Animals occupy sleeping structure	1.13 (1.03, 1.24)	*0.95 (0.92, 0.97)*	*0.92 (0.86, 0.99)*		

Odds ratios (95% confidence intervals)

^A^Adjusted for net age; italic indicates associations with better adherence, underline indicates associations with worse adherence, White space indicates no association. N/A indicates not applicable

As compared to those properly stored during the day, nets that remained hanging were more likely to belong to a household with high confidence to prevent malaria and less likely to have been used by a child < 5 years in both sites (Table 5). This suggests that households with a sense of self-efficacy to prevent malaria were less likely to properly store bed nets, and nets used by young children were stored properly during the day. Other factors were positively and negatively associated with storage practices, but different across sites (Table 5).

Households that learned about net care from community events, that shared a sleeping structure with animals, and that had nets used by adult males tended to adhere to washing frequency recommendations (Table 5). It was hypothesized this difference could be due to housing structural factors where dustier environments may be associated with more frequent washing. However, no association was found between having dirt floors and washing behaviours in either site (Table 5).

There was a remarkable difference in drying practices between sites. In the lowlands, 32% of nets were dried in the sun compared to only 4% in the highlands (Fig. 2). Drying in the shade may not be feasible for individuals depending upon the environment surrounding their compound, so the study examined whether amount of shade was associated with drying practices, but found no association, though households in the lowlands had less shade compared to households in the highlands (Table 3). Interestingly, nets improperly dried in the sun were less likely to belong to a household with high knowledge of proper drying practices, indicating that increasing knowledge may positively influence this practice. Source of net care knowledge was similarly associated, suggesting that community events were effective in their messaging regarding proper drying practices (Table 5).

## Discussion

Net care adherence scores were generally low (adherent) in both the highlands and lowlands of western Kenya. Nets had median adherence scores of 2 and 4, respectively, with 0 being the optimal score out of a possible 14. However, there are a few key care behaviours that could be targeted for improvement in the lowlands. Net storage, washing frequency, and drying are the key areas for interventions to address.

Tying up nets or storing them in a safe space while not in use during the day prevents physical damage. This practice may also increase the amount of time a net appears clean, which may lead to less frequent washing. Focus group participants in Senegal reported that tying up nets during the day is a best practice for preventing damage, and removing the net and storing it in a safe space is also a good practice particularly when children are likely to pull on hanging nets [19]. A ‘trials of improved practices’ study in the Peruvian Amazon region found that some households that did not remove their nets during the day developed ways of tying the nets and covering them with plastic so they could remain hanging during the day and still be protected from damage [31]. In this study, variables associated with leaving a net hanging rather than tied up during the day were varied, and span attitudes and environmental factors in both the highlands and lowlands. This suggests that there is no one factor that influences net storage behaviour, but a combination of various factors do. Harvey and others reported 15 different reasons a household left a net hanging, the most common of which included lack of time, someone rests under the net during the day, feeling lazy, or forgetting to remove the net [31], indicating that it is inconvenient and time consuming to remove nets during the day. Tying up nets during the day is less labor and time intensive, and prevents damage compared to nets left hanging over sleeping spaces during the day.

Frequent washing can degrade both the physical and chemical integrity of LLINs, so current recommendations are to wash LLINs at most every 3 months [16]. Many nets are washed more frequently than the current recommendations. In the lowlands nearly 80% of nets were washed < 3 months prior to the survey. Other studies have found variable washing frequencies spanning from 1.5 washes per year on average in Uganda [32] to up to 8 washes per month in Mali [33]. Additionally, 31% of nets were washed more frequently than the recommended practice in a Kenyan study conducted among the southern coastal districts [34]. These differing wash frequencies across studies make generalizability difficult, however the reasons for doing so seem to be less related to knowledge of preferred practices and more related to social and structural factors. Nets simply get dirty quickly, and households (due to personal or social acceptability reasons) prefer to wash nets when they are dirty [18, 21, 22, 31, 33, 35]. Similarly in the present study, washing frequency was associated with environmental factors, and not with knowledge and attitudes like net care knowledge, perception of malaria risk, or confidence in ability to prevent malaria. Although there is great variation across studies, it is important to monitor the practice and understand why households perform variable washing practices due to the significant implications for net longevity.

Soap type and net brand may be modifying factors with washing frequency depending on the pH level of the soap. Bar soaps and mild detergents cause less damage to insecticide levels on nets compared to bleach. In this study, no nets were reported to be washed with bleach, though the brands or pH levels of soaps and detergents used could not be determined. A washing trial measuring insecticide residual after set time intervals of 6 or 12 months following net distribution in Uganda found that nearly all (99.6%) of nets in the region were washed with mild soap (pH 9–10) and the remaining nets washed with detergent [36] while this study found a greater proportion of nets were washed with detergent (21.2% of nets in the highlands and 53.5% of nets in the lowlands). With respect to adherent soap type practices, the Ugandan study found that insecticide integrity differed by net brand and net generation within net brands [36]. Washing nets with more stringent detergents rather than mild soap may impact insecticide integrity. A study in western Kenya tested insecticide effectiveness by measuring how many mosquitoes were able to feed through nets and how many mosquitoes died after net exposure after 5, 10, and 15 washes with a detergent commonly used in rural areas [37]. Even after only 5 washes with detergent, up to 17.6% of mosquitoes were able to feed through nets and up to 83.3% of mosquitoes remained alive 24 h after net exposure depending on net brand [37]. Though this study did not compare mosquito ability to feed or mosquito mortality after exposure to nets washed without detergent, this suggests that while net brand also plays a role, insecticide integrity is noticeably compromised even after 5 washes with detergent [37]. For consistent messaging, recommendations should continue to emphasize the use of no or very mild soaps for net washing, regardless of net brand.

Drying nets in the sun is not recommended because direct sunlight degrades insecticides [4]. As many nets are dried in the sun in the lowlands, proper net drying is a key net care component to be addressed. Another study in Kenya found that 64.9% of nets were dried in the sun [38], and nets were observed drying in direct sunlight in Uganda as well [39]. These results suggest that knowledge and attitudes, particularly a lack of knowledge of appropriate net drying influence this particular care practice. Learning net care from a community event was associated with better net drying adherence, suggesting education campaigns geared toward community settings may be effective in improving net drying practices in the lowlands sites.

### Implications for interventions

Net washing frequency, use of soap, and drying location affect net longevity [22, 32, 39–43]. Other studies have found that LLINs do not last as long as intended in the field, often in poor condition after 1–2 years of use [10, 14]. While there is variation among studies in net longevity following distribution campaigns [44], many nets are lost to attrition or are damaged before the universal 3 year life span [4].

Because LLINs involve multiple care and repair practices, and each practice is influenced by multidimensional factors spanning attitudes and environmental factors, intervention strategies should be variable and informed by context- and location-specific care and repair practices [45]. Because of the varied and at times non-existent associations between knowledge of net care items and adherence to care practices, education interventions alone are unlikely to markedly improve bed net care and repair. Attitude factors are likely the most difficult to change as they are related to cultural and personal belief systems. Thus, successful solutions aimed at improving care and repair should particularly involve environmental interventions. Some possibilities to explore include providing materials to assist with tying nets up during the day if household structures do not easily permit net folding or tying. A recommendation by Harvey and others is to design nets to be easily pulled up so they can remain hanging, yet be more protected from damage [31]. Because many nets were left hanging during the day in the present study, these results support that this suggestion could prevent physical degradation without necessitating behaviour change. Furthermore, consistent and complete care and repair messaging provided from any place where a net is distributed (particularly clinics and distribution events) is recommended. Demonstrations of proper care and repair activities may be particularly effective.

## Limitations

Net care and repair practices are likely quite different between communities, regions, and countries, making comparisons and generalizability between sites and other studies difficult. This highlights the importance of LLIN monitoring after distributions in different locations to track and respond to local trends of net ownership, use, and maintenance. The President’s Malaria Initiative (PMI) hosts a LLIN durability monitoring repository (http://www.durabilitymonitoring.org) to track LLIN field durability among different locations [5] and is an excellent resource for comparing results across studies.

“*Net condition”* was classified based on standard images provided in the survey forms (see Additional file 1), but this was not a standard method of measuring net condition. Using a proportional hole index would have been a standard and more comparable method of collecting this information, but was unavailable for these analyses. Additionally, net repair practices were not measured, so this could not be included in the net care adherence score.

To describe net storage during the day, nets that were removed during the day were categorized as a moderately adherent practice. However, it is unknown where the nets were placed during the day, so there is potential for misclassification if these nets are removed to a space that makes them prone to damage rather than a safe storage space.

It is possible that the person reporting for the household may not be the same person who cares for the nets, which could lead to misclassification. However, there were options for “don’t know” in survey items, hopefully limiting this occurrence. Additionally, social acceptability bias is possible as individuals might report better care practices than are actually performed, particularly if they have high knowledge of net care. Another limitation is the inability to distinguish between activities conducted at community events and net distributions. There may be misclassification if some individuals consider a distribution to be a community event. This is unlikely to change the results, however, as in the lowlands almost no one reported receiving education during a distribution and both community events and distributions in the highlands were associated with better adherence.

## Conclusions

There was high adherence to recommended net care practices in western Kenya. Net care and repair is influenced by various attitudes and environmental factors. There is little evidence that behaviour change is needed in the highlands sites of western Kenya. In the lowlands, promoting methods for tying up nets during the day, coupled with messaging to emphasize infrequent washing and drying nets in the shade may be an appropriate intervention. If there are concerns about net longevity following a distribution, the need for behaviour change interventions should be assessed in local contexts to target specific practices and the driving forces underlying those practices.

## Additional file

**Additional file 1: Figure S1.** Observational overall bed net condition. The field team was instructed to compare each bed net to these standard images to classify overall netcondition as Excellent, Good, Fair, or Poor.

## Data Availability

The datasets used and/or analysed during the current study are available from the corresponding author on reasonable request.

## Abbreviations

ITNs: insecticide treated nets
LLINs: long lasting insecticide treated nets
PMI: President’s Malaria Initiative
KEMRI: Kenya Medical Research Institute
GEEs: generalized estimating equations
