# Supplementary material for: Bed net care practices and associated factors in western Kenya
Source: Malar J. 2019 Aug 14;18:274. doi: 10.1186/s12936-019-2908-6 (PMC6694604; doi:10.1186/s12936-019-2908-6)
Supplement: Supplementary file 1 — Additional file 1: Figure S1. Observational overall bed net condition. The field team was instructed to compare each bed net to these standard images to classify overall netcondition as Excellent, Good, Fair, or Poor. [file 12936_2019_2908_MOESM1_ESM.pdf]

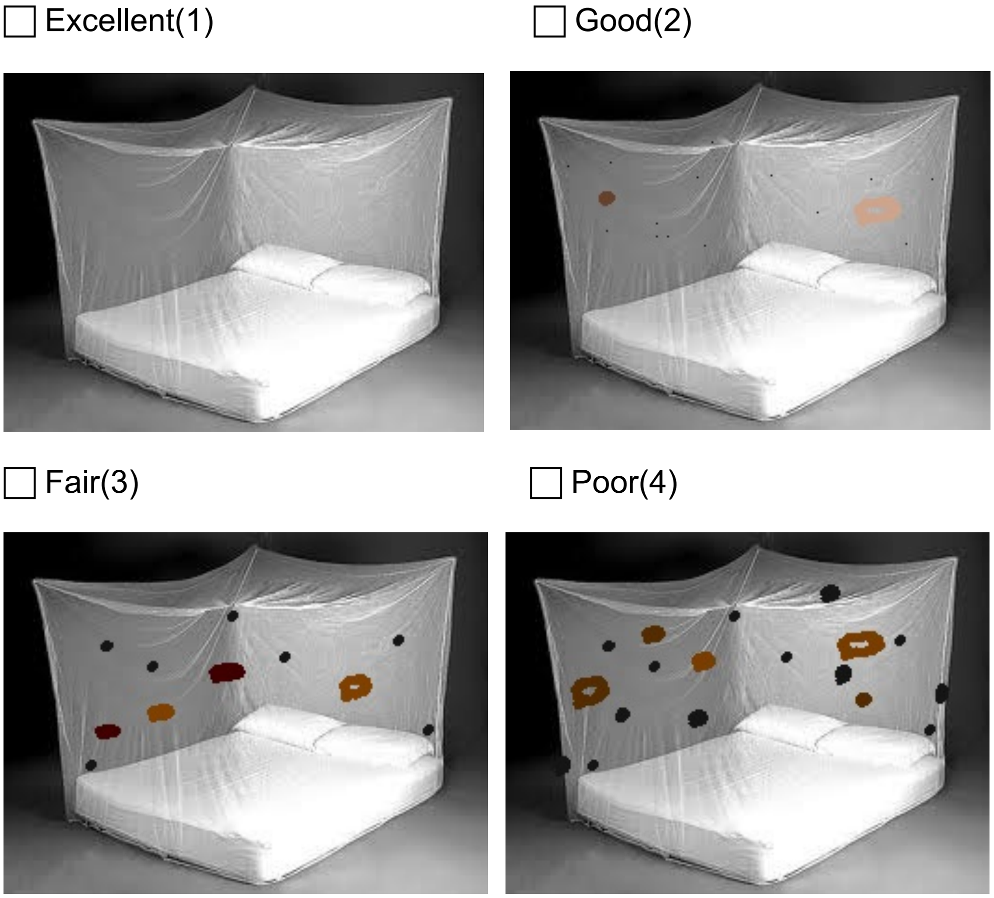


**Additional File 1: Figure: S1.** Observational overall bed net condition. The field team was instructed to compare each bed net to these standard images to classify overall net condition as Excellent, Good, Fair, or Poor.
